# A Comparison of Detour Behaviors in Some Marine and Freshwater Fish Species

**DOI:** 10.3390/ani14172572

**Published:** 2024-09-04

**Authors:** Davide Potrich, Chiara Orsini, Gionata Stancher, Greta Baratti, Valeria Anna Sovrano

**Affiliations:** 1 Center for Mind/Brain Sciences (CIMeC), University of Trento, 38068 Rovereto, Italy; greta.baratti@unitn.it; 2 Institute of Psychology, University of Innsbruck, 6020 Innsbruck, Austria; chiara.orsini@uibk.ac.at; 3 Rovereto Civic Museum Foundation, 38068 Rovereto, Italy; stanchergionata@fondazionemcr.it; 4 Department of Psychology and Cognitive Science, University of Trento, 38068 Rovereto, Italy

**Keywords:** fish, cognition, detour behavior, insight, object permanence, problem solving, route planning

## Abstract

**Simple Summary:**

Detour behavior refers to the ability to reach a goal object that is not directly accessible due to an obstacle (opaque or transparent) by circumventing it. It varies among species, suggesting that environmental adaptation may drive insight behavior. Some species of marine and freshwater fish were placed in a corridor with social stimuli at the end, not directly accessible due to an opaque barrier. Two symmetrical apertures positioned midline in the corridor allowed the fish to temporarily abandon the view of the goal and attempt to circumvent the barrier. All fish showed the ability to move around an interposed obstacle. This is the first evidence of detour behavior in marine fishes within the “four-compartment box task”, while results in freshwater fishes confirmed previous evidence. The comparable performance of marine and freshwater fishes suggests similar selective ecological pressure even in different aquatic habitats (e.g., sea and freshwater basins). Moreover, different exploratory behaviors emerged between correct and incorrect compartments (particularly in *Danio rerio*), providing evidence for a possible mental representation or “permanence in existence” of the goal while performing the obstacle circumvention, as opposed to a more parsimonious idea suggesting that the detour ability emerges only from primitive forms of exploratory behavior such as taxis.

**Abstract:**

Evidence of detour ability to reach a salient goal in marine fishes (*Chromis viridis*, *Chrysiptera parasema*, *Dascyllus aruanus*) and freshwater fishes (*Xenotoca eiseni*, *Danio rerio*) has been observed using a “four-compartment box task” with an opaque barrier. The first experiment investigated this ability in marine fishes (*Chromis viridis*, *Chrysiptera parasema*, *Dascyllus aruanus*). Fish were placed in a four-compartment box, with social stimuli not accessible due to an opaque barrier. Two symmetrical apertures midline in the corridor allowed the fish to temporarily abandon the goal’s view and attempt to circumvent the barrier. Marine fish showed searching behavior in the two compartments near the social stimuli. In the second experiment, the detour abilities of a marine fish (*Dascyllus aruanus*) and two freshwater fishes (*Xenotoca eiseni*, *Danio rerio*) were compared using a modified version of the apparatus, with elongated compartments continuing further from the obstacle barrier and social stimuli. This enabled the evaluation of the dependence on effective distance to achieve the social goal. Both marine and freshwater fish exhibited detour skills. Additionally, *Danio rerio*’s differential spatial explorations inside compartments supported an active interest in searching for conspecifics, suggesting possible social object permanence retention. Overall, these results highlight the ecological salience of detour skills in fishes, irrespective of species-specific adaptations.

## 1. Introduction

Detour behavior is the ability to reach a goal stimulus by moving around an obstacle, whether opaque or transparent, that is interposed between the subject and the stimulus [1]. Such a fundamental problem of detouring around obstacles to reach a goal can be assessed to study multiple cognitive competencies [2]. In order to solve it, the spontaneous, attractive behavior towards the stimulus must be inhibited, together with planning a route, circumventing the obstacle by moving far from it before reaching the stimulus and, therefore, probably keeping in mind the object to be reached even when no longer visible. The first description of detour abilities dates back to observations carried out by Köhler [1], who reported in various animals (chimpanzees, dogs, and chicks) the ability to detour wire fences or to retrieve an object thrown through a window. Köhler interpreted those behaviors to be due to an “insight” since the animal must have a perception of the entire problem before solving it via a detour. After Köhler’s initial observations, detour abilities have been widely studied in several animal species over the years, providing useful information on how performance varies among species and highlighting potential factors that can affect its solving ([2], for a complete review).

Although different methods and apparatuses have been used, detour tasks can be grouped into two main categories, depending on whether the goal is continually visible or only initially visible behind a barrier but not during the detour. In the “constantly visible goal” scenario, the barrier to be circumvented is usually a transparent or semitransparent obstacle, such as a shaped barrier (e.g., V-shaped: [3]; U-shaped: [4]; I-shaped: [5]; L-shaped: [6]; J-shaped: [7]), a transparent cylinder accessible through one of the ends, e.g., [8,9,10]), or a transparent box with only one open side (e.g., [11]). These tasks are excellent for measuring inhibitory control skills. The visible reward behind the transparent barrier is a strong trigger that must be inhibited in order to adopt a detour behavior. Several lines of evidence have shown that visibility of the reward could affect performance: the more visible the goal behind the barrier (e.g., a plexiglass barrier is clearer than a grid), the more difficult it is for the animal to exert inhibitory control (e.g., [3,12,13]). The proximity of the stimulus is another factor that may influence performance (i.e., the closer the goal, the more difficult the inhibition) (e.g., [12,14]). Moreover, perceiving an obstacle as such is essential for the commencement of a detour. For example, chicks that see the goal through a transparent or a vertical bar barrier take longer to solve the task than when the bars are horizontal due to a weaker perception of them as an obstacle (e.g., [12,15]).

When the goal is not constantly visible throughout the detour response, the subject can observe the goal only from the barrier, becoming not visually accessible once the animal detours due to the presence of an obstacle. This setup is typically called “initially visible goal detour,” and it is mostly used for investigating cognitive aspects other than inhibitory control, such as route planning and working memory. Since detouring the obstacle causes a temporary cessation of sensory contact with the goal, the subject needs to retrieve a mental representation of the goal from working memory. If the animal cannot maintain a (presumed) hidden object’s permanence, its recovery is theoretically not possible. Studies suggesting animals’ understanding of object permanence have been described in primates (e.g., [16,17]), other mammals (e.g., [18,19,20]), birds (e.g., [21,22]), and fish [23]. An efficient setup designed for evaluating detour ability in case the goal is only initially visible is the so-called “four-compartment box task” developed by Regolin and colleagues [24] for chicks. After observing the goal through a barrier, the chick was required to move far from it and circumvent the obstacle, losing visual contact with the stimulus and directing its choice towards one of four compartments, with only two leading the individual toward the goal. Chicks properly approached the compartments leading to the goal stimulus, avoiding the other two far from it (see also [12,15,25]).

Since the goal is only visible at the beginning and not during the detour action, it has been proposed that animals must have a mental representation of the goal while performing the obstacle circumvention [15,26]. Nevertheless, the nature of such a representation in solving the detour problem has been questioned in favor of a more primitive behavior guided by environmental and sensorimotor affordance, such as wall-following movement or visual scanning (e.g., [27,28]). Interesting evidence supporting a less demanding cognitive strategy has been shown by Walker and Miglino [29] in a study conducted with simulated robots. The authors replied to the results obtained by Regolin and colleagues [24] in chicks, showing that artificial agents make detours based on motor inputs detected by the proximity sensory feedback, without any internal pre-programmed representation of the goal. Instead, they utilized simple exploratory behavior and taxis [29,30]. Nevertheless, this would not exclude that animals can retain a mental representation of an object but suggests that such a mental image is not required for solving the task.

The detour ability has been described as a cognitive tool shaped by selective environmental pressures. Barash [31] reported a clear example of different performances between dogs and squirrels when circumventing a pole to reach a food site: dogs were not able to solve the task, while squirrels detoured around the pole immediately. This difference has been ascribed to the phylogenetic history of adaptation to the environment, where squirrels, unlike dogs, frequently need to circumvent trees or branches to reach a salient goal. Further evidence comes from Zucca and colleagues [32], who observed that detour performance could vary among bird species that inhabit different ecological environments. Among the species studied, quails (*Coturnix* sp.) and herring gulls (*Larus cachinnans*) performed the task more successfully than canaries (*Serinus canaria*), which were unable to undertake a detour to reach the goal. This divergence has been attributed to the role of different adaptation niches (i.e., terrestrial or aerial environment), suggesting that canaries (aerial species) do not solve the detour problem because most obstacles encountered can be avoided simply by flying over them.

A comparison among species belonging to different environments has also been made in four species of teleost fish. Sovrano and colleagues [23] observed detour behavior in freshwater fishes *Danio rerio*, *Xenotoca eiseni*, *Carassius auratus*, and *Pterophyllum scalare* in a task with an “initially visible goal” (i.e., the four-compartment box task). They reported that all four species predominantly approached the compartments close to a previously spotted social goal stimulus (i.e., a group of conspecifics). In this case, the absence of differences suggests that similar ecological pressures and needs are shared among these species, building on other evidence in freshwater fishes revealing the circumvention of a transparent or semitransparent barrier in a “constantly visible goal” condition as well [10,33,34,35,36,37,38,39]. Despite the vast number of fish species already studied, it remains poorly investigated whether other fishes adapted to very different habitats, such as marine fish, show consistent behavioral patterns for detouring. In the tropical marine fish *Labroides dimidiatus*, detour skills have been successfully studied under learning conditions and progressive increase of the complexity or ecologically relevant contexts of the task, showing inhibitory control abilities in learning to detour a barrier to achieve a non-social reinforcement (e.g., food reward) [40,41].

Interestingly, various behavioral patterns have been described among marine and freshwater fishes in other non-detour tasks. A strong preference for using the right eye when inspecting social stimuli from a mirror has been found in marine fishes *Acanthurus triostegus* [42] and *Myrispristis pralinia* in recognition of conspecifics [43]. The preference for the right eye is intriguing since it contrasts with the preferential use of the left eye observed during the inspection of mirror self-images in freshwater fishes and amphibians, and which conforms with the key role played by the right telencephalon hemisphere in social cognition [44,45,46,47,48,49,50]. Such differences suggest that different laterality patterns may be associated with habitat preference (e.g., freshwater vs. marine) or adaptation history and driven by ecological pressures or phylogenetic constraints [42].

It is of particular interest to investigate and compare whether two aquatic habitats, which are strongly different from an ecological point of view, can affect performance in the detour test of phylogenetically distant fish species in the presence of a shared element, i.e., the presence of obstacles. For this reason, we selected three species of marine Damselfish associated with coral reef habitats and two species of freshwater fishes living in stagnant water with submerged vegetation. We hypothesize that the presence of obstacles, regardless of their nature and characteristics, played a role in selecting (or maintaining) the cognitive ability to perform the detour test in fishes.

Detour abilities in a “four-compartment box task”, with an opaque barrier and consequent loss of visibility of the social target during the circumvention but without learning over time, have only been observed in freshwater fish species [23]. Thus, the present study first aimed to explore detour behavior in three marine fish species (*Chromis viridis*, *Chrysiptera parasema*, and *Dascyllus aruanus*) in the typified four-compartment box task, as previously addressed in freshwater fishes [23] and birds [24,32], allowing to directly compare the performance among species. These marine coral reef fish are gregarious species, benefiting from schooling in successful prey capture and defense against predators [51,52]. The groups can be more or less numerous depending on territorial habits and aggression, greater in *C. parasema* [53].

In the four-compartment box task, animal preferences are typically measured and completed as soon as the subjects enter one of the four opaque compartments, which are immediately available after the obstacle circumvention, without observing how animals behave in the chosen compartment. Therefore, the second aim of this study was to investigate how fishes behave when a larger circumvention is required and whether different exploratory patterns are present when the chosen path leads toward the correct or incorrect compartment (i.e., the compartment close to or far from the goal, respectively). This was tested in a second experiment, using a bigger apparatus in which the obstacle circumvention led to long compartments that reached and overcame the exact spatial position of the goal stimulus at a certain distance from the barrier, allowing observation of potential differences regarding the time required to perform the choice and activity inside the chosen compartment. The hypothesis tested here was whether choosing the correct compartments (detour close to the goal) could correlate with a distinctive decision time and/or exploratory routine when the correct or incorrect compartment was selected, thus providing important evidence regarding the actual intention to approach the goal.

## 2. Materials and Methods

### 2.1. Experiment 1—Detour Behavior in the Standard Four-Compartment Box

Forty-eight marine fishes (*Chromis viridis*, N = 16; *Chrysiptera parasema*, N = 16; *Dascyllus aruanus*, N = 16) took part in Experiment 1. All the marine fishes were not sexed due to the unclear sexual dimorphism.

Fishes were provided by a local store (“Acquario G” Trento, Italy) and housed in 25 L tanks enriched with gravel and synthetic plants to provide them with a comfortable environment. The acclimatization period in the animal house was at least one month. The quality and cleanliness of the marine water and experimental apparatuses were maintained through oxygenators (Air 275 R Plus, SERA, Heinsberg, Germany), ceramic rings, and a stream pump (Koralia Evo nano 900, HYDOR, Bassano del Grappa, Italy; flow rate 900 L/h). The marine water temperature was kept at 28 °C, and the salinity level was maintained stable, with water density between 1018 and 1020 g/dm^3^. Fishes were fed three times per day with dry food (Ocean Nutrition flakes for marine fishes).

**Figure 1:**
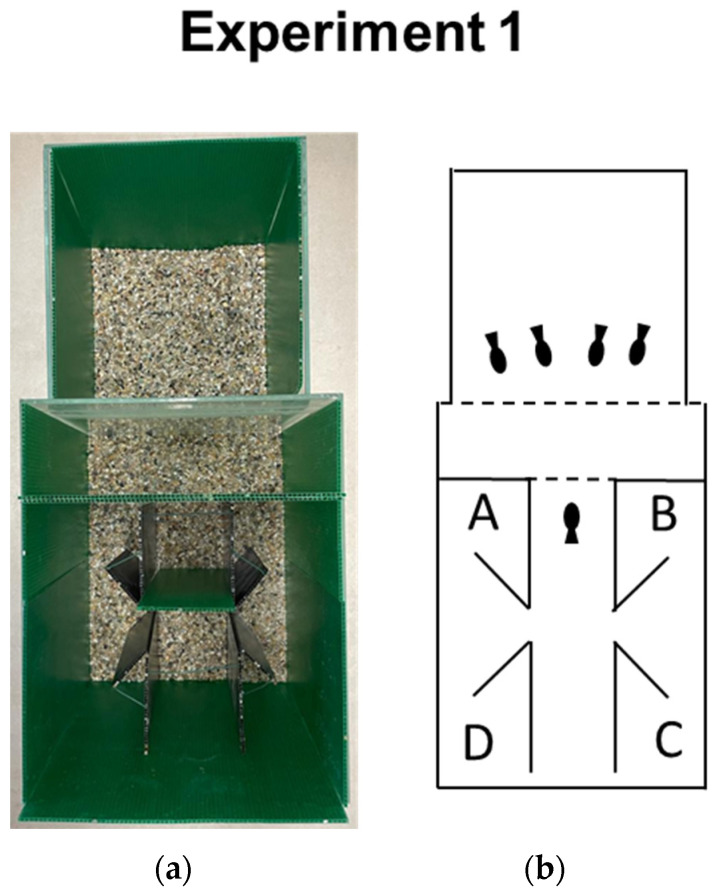
(**a**) Photograph from above of the apparatus used in Experiment 1, with two adjacent glass tanks covered with dark green plastic panels. The smaller tank (on the top) housed four adult fish as social attractors for the experimental animal, instead located in the larger tank (on the bottom); (**b**) Schematic representation of the experimental apparatus in the standard four-compartment box (Experiment 1). The two correct and incorrect compartments are labeled as A–B and C–D, respectively, and were placed at the ends of the corridor. A and B were located close to the grid (dotted line), through which social attractors could be observed, while C and D were on the opposite side of the larger experimental tank. All the corridor’s sides and partitions were identical and made of opaque plastic material, not allowing anything to be seen outside (blind compartments) (solid lines).

The experimental apparatus was a replica of the one implemented by Sovrano et al. [23], consisting of two adjacent transparent glass tanks (35 × 30 × 25 cm and 25 × 25 × 25 cm) facing each other (Figure 1). The larger tank housed the experimental subject, while the smaller one housed a group of four conspecifics, acting as a socially attractive stimulus. The walls of each tank were internally covered by dark green polypropylene sheets (Poliplak^®^), except for the shared adjacent glass sides, through which the experimental subject could observe the conspecifics. The floor of each tank was covered with gravel, and the water level was 19 cm in height. The same level of gravel and water within the two tanks was kept in order to guarantee visual continuity, which is essential for the detour task.

Inside the larger tank, there was a corridor made of two black plastic walls (24 cm length, 9 cm width, 22 cm height). At one end of the corridor, a dark green panel with a rectangular aperture (5 × 14 cm) with a thick grid (0.2 mm) allowed the experimental subject to observe from an “observation area” (9 × 9 × 25 cm) a group of four conspecifics (15 cm far away), which were hosted within a compartment made of green plastic (25 × 25 × 4 cm), located in the smaller tank. At the midline of the corridor, two symmetrical apertures (4 cm) allowed the fish to go outside the corridor and enter one of the four compartments, which were delimited by diagonal partitions (5 cm long and 22 cm high, 45° inclined with respect to the corridor’s side). For this detour task, the two compartments closest to the obstacle (compartments A and B) were coded as correct, while the two located in the opposite direction from the conspecifics’ tank (compartments C and D) were coded as incorrect. All the corridor’s sides and partitions were identical and made of black (the partitions) and green (the surrounding) plastic material, not allowing anything to be seen outside (blind compartments).

The experiment took place in a darkened and acoustically isolated room. The apparatus was lit centrally from above (30 cm high) by a 3W LED bulb. Fish behavior was recorded with a webcam (LifeCam Studio, Microsoft) positioned 30 cm above the apparatus.

In each trial, using a removable sliding dark green panel, the experimental subject was gently confined into the “observation area” for 5 min, from which it could observe the social group in the adjacent smaller tank (see Figure 1). At the end of the observation phase, the removable panel was slid up, allowing the fish to leave the observation area and approach one of the four compartments. The fish was free to enter one of the four compartments within a 10 min time limit. The choice was considered completed when the fish entered entirely into the chosen compartment (indicated as A, B, C, or D). Once the choice was made, the trial ended, and the fish was placed back into the observation area for the subsequent trial. Each fish performed four trials, divided into two daily sessions (two trials per day). At the end of the second trial, the fish was placed back into the home tank. The day after, the fish was exposed to the same procedure as the day before in order to have a total of four valid choices per subject, according to the procedure already adopted in previous studies [23,24]. In the event that the subject did not choose one of the four compartments in the maximum time allowed, the trial was repeated. In case of two consecutive null trials, the fish was placed back into the home tank, observed the next day, and possibly dismissed from the experiment after two null daily sessions.

Data were analyzed using IBM SPSS Statistics software (Version 27).

Choices of compartments A or B were collapsed into the category “correct”, while choices of compartment C or D were collapsed into “incorrect’. Considering the total choices collected over the two days, we conducted an analysis of variance (ANOVA) with species as between-subjects factors; and sectors (A–B vs. C–D) and day (1st day vs. 2nd day of test) as within-subjects factors. To estimate the effect sizes, partial η^2^ as the index for ANOVA was reported. Differences between the correct compartments (A vs. B) and the incorrect ones (C vs. D) were analyzed through a paired Student’s *t*-test, reporting Cohen’s *d* as an effect size measure.

A Kruskal–Wallis test was used in the analysis, limited to the first choices, in order to detect statistical differences between species when choosing the two correct compartments A–B. Data were then analyzed using a χ^2^ test to detect whether the choice of the correct compartments was significant.

### 2.2. Experiment 2—Detour Behavior in the Enlarged Four-Compartment Box

Sixteen naïve marine fish (*Dascyllus aruanus*) and forty-eight freshwater fish (*Xenotoca eiseni*, N = 24; *Danio rerio*, N = 24; half females and half males) took part in Experiment 2. The marine fish were not sexed due to the unclear sexual dimorphism. The two freshwater species were those already successfully observed at the standard “four-compartment box” detour task [23].

Freshwater fishes came from our laboratory stock and were housed in 25 L tanks enriched with gravel and synthetic plants to provide them with a comfortable environment. The freshwater temperature was kept at 26 °C and filtered by an external pump (Niagara 250, WAVE). Fishes were fed three times per day with dry food (Sera Vipan for freshwater fishes).

As in Experiment 1, fish were allowed to observe a group of four conspecifics through a grid without the possibility of joining it directly. In sexed species, the group of four conspecifics consisted of females. After an observation phase, the subjects were allowed to explore the apparatus and approach one of the four corridors, losing sight of the social attractors. But differently from Experiment 1, here, fish were required to adopt a larger detour and enter one of four elongated compartments that could extend in the direction of the social group (compartments A and B) or the opposite direction (compartments C and D). Besides observing detour ability in a larger apparatus, it was checked whether fish behavior was different when the correct or incorrect compartments were chosen by analyzing the time needed to complete the trial and the depth of exploration in the selected compartment.

**Figure 2:**
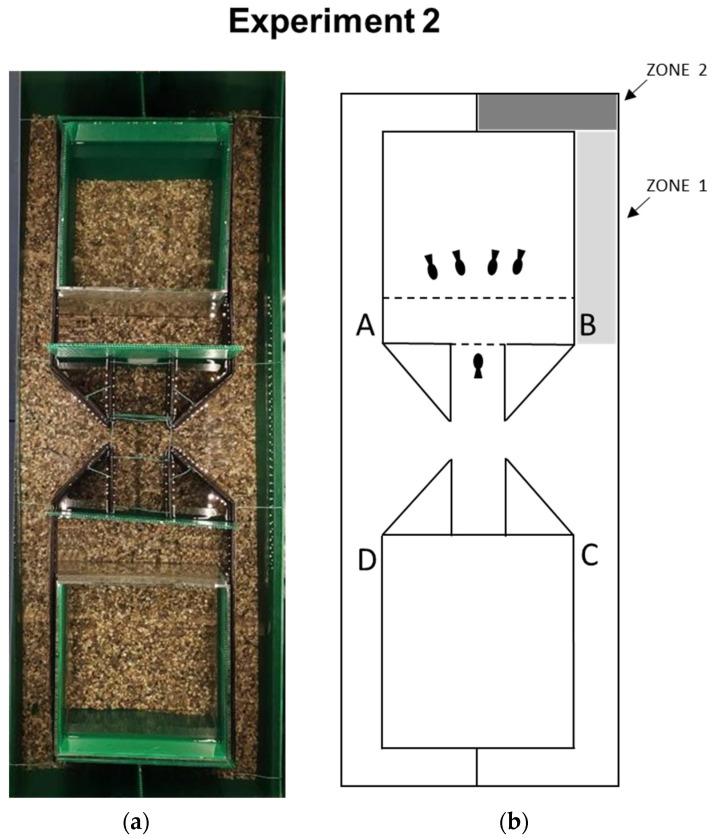
(**a**) Photograph from above of the apparatus used in Experiment 2, with two identical glass tanks covered with dark green plastic panels. The two tanks could alternatively house four adult fish as social attractors for the experimental animal instead located in the corridor on the midline of the apparatus. (**b**) Schematic representation of the experimental apparatus in the enlarged four-compartment box, exemplifying an experimental session. The two correct and incorrect compartments are labeled as A–B and C–D, respectively, and could be reversed between the two daily testing sessions, counterbalancing among the subjects. This enables the exclusion of any potential bias due to the apparatus. In this way, the grid through which social attractors could be observed could be reversed between daily trials, obscuring it with a removable panel (solid line) or making it accessible (dotted line). The colored areas delineate the different choice zones (zone 1, the effective distance of social attractors, and zone 2, a greater distance than the position of social attractors).

The experimental apparatus was a modified version of the one used in Experiment 1. The experimental apparatus consisted of a large tank (120 × 45 × 50 cm) covered from the inside with a green propylene sheet (Poliplak^®^). The structure of the observation phase, the central corridor, and the tank housing the social group were maintained identically as in Experiment 1. The critical difference introduced here is the lengths of the barriers and compartments (Figure 2). At the midline of the central corridor, the four diagonal partitions were prolonged (12 cm long and 22 cm high, 45° inclined with respect to the corridor side). At the end of the inclined partitions, the extensions continued parallel to the central corridor, creating four long identical compartments (45 cm long, 22 cm high, 6 cm wide) finishing in a dead-end after a 90° turn. The choice selection criterion was that the body of the fish fully enters one of the channels. In order to exclude any potential bias due to the apparatus, the position of the observation area and the social group were counterbalanced between opposite sides of the apparatus. This was possible thanks to the use of two opposite tanks (25 × 25 × 25 cm), one of which housed the social group, and the use of interchangeable panels allowing swapping of the observation area position.

In the evaluation of the detour task, the two compartments closest to the obstacle (A and B) were coded as correct, while the two located in the opposite direction than the conspecifics’ tank (C and D) were coded as incorrect. Two LED strips (60 cm long, 17 W, 1600 lm, 2900–300 K cool white), which were positioned parallel to the corridor at a distance of 50 cm, provided illumination. Fish behavior was recorded using a webcam set above the apparatus (60 cm high).

The observation phase was the same as described in Experiment 1. At the end of the observation phase, the subject was allowed to explore the apparatus. A choice was considered completed when the fish crossed the threshold into one of the four compartments. For each fish, the time required to exit from the observation area (i.e., time 1), the time elapsed after exiting the observation area, and the time at which the choice of the compartment was made (i.e., time 2) were measured. The trial was considered concluded as soon as the fish, after choosing a compartment, reached the bottom of the compartment or went back and exited from the compartment entrance: these were coded as two different exploratory behaviors, namely “exploration before the end of the compartment” (i.e., zone 1) or “exploration until the end of the compartment” (i.e., zone 2) (see Figure 2b). Once the choice was made, the trial ended, and the fish was placed back into the observation area for the next trial. Each fish performed a total of four trials, divided into two daily sessions (two trials each). The position of the social stimuli (left or right tank) and the correct/incorrect compartments varied between day one and day two and were counterbalanced among the subjects.

Data were analyzed using IBM SPSS Statistics software (Version 27).

Choices were collapsed into the categories of correct and incorrect, as in Experiment 1. Considering the total choices collected over the two days, we conducted an analysis of variance (ANOVA) with species and sex as between-subjects factors; and sectors (A–B vs. C–D) and day (1st day vs. 2nd day of test) as within-subjects factors. To estimate the effect sizes, partial η^2^ as the index for ANOVA was reported. Differences between the correct compartments (A vs. B) and the incorrect ones (C vs. D) were analyzed through a paired Student’s *t*-test, reporting Cohen’s *d* as an effect size measure.

A Kruskal–Wallis test was used in the analysis, limited to each fish’s first choice, in order to detect statistical differences among species when choosing the two correct compartments A–B. Data were then analyzed using a χ^2^ test to detect whether the choice of the correct compartments was significant.

The correlation between the “performance accuracy” index and the “compartment exploration” index or “time” was analyzed using a Pearson correlation coefficient.

## 3. Results

### 3.1. Experiment 1—Detour Behavior in the Standard Four-Compartment Box

**Figure 3:**
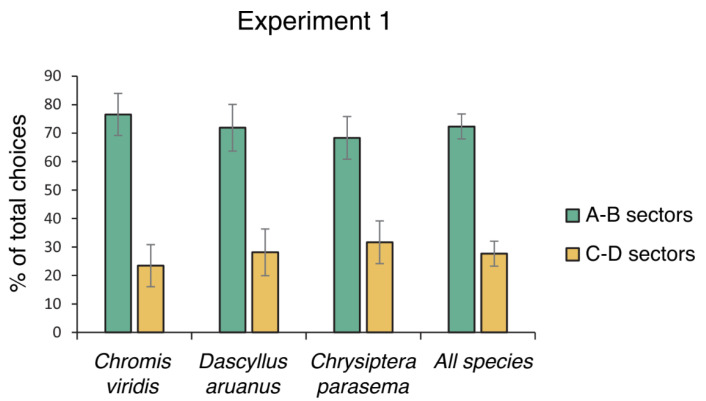
Percentage of total choices (mean ± standard error of the mean [SEM]) for the correct compartments close to the social goal (A–B) and the incorrect compartment far from the social goal (C–D), considering species separately and together.

Only 1 of the 16 *Chrisiptera parasema* failed to exit the corridor within 10 min for multiple trials, showing a freezing reaction. All the other fish completed the four test trials (Figure 3). Cumulative choices for the compartments visited on the total of the four trials were analyzed using an ANOVA, considering the compartments (correct: A–B vs. incorrect: C–D) and days (1st day vs. 2nd day of the test) as within-subject factors, while the species (*Chromis viridis*, *Chrysiptera parasema*, *Dascyllus aruanus*) were included as between-subject factors. The analysis revealed a significant effect of compartments (F(1,44) = 24.955, *p* < 0.001, $\eta_{p}^{2}$ = 0.362), while no other statistical effects were found among species or days and their interaction with compartments (compartments × species: F(2,44) = 0.284, *p* = 0.754; compartments × days: F(1,44) = 0.097, *p* = 0.765; compartments × days × species: F(2,44) = 2.624, *p* = 0.087). A paired Student’s *t*-test, applied to compare the correct compartments A vs. B and the incorrect compartments C vs. D did not reveal statistically significant differences (total choices: A vs. B: t(46) = 1.855, *p* = 0.070, Cohen’s *d* = 0.271); C vs. D: t(46) = 0, *p* = 1.00, Cohen’s *d* = 0), highlighting a balance of choices between the two pairs of compartments (correct vs. incorrect).

Analysis limited to the first choices using a Kruskal–Wallis test revealed no statistical difference among species in choosing the two correct compartments A–B (H(2) = 0.632, *p* = 0.729). A general χ^2^ test showed a preference for the correct compartments over the incorrect ones (χ^2^ = 9.383, *df* = 1, *p* = 0.002).

Overall, the results of Experiment 1 showed that all the marine species considered seemed able to detour an obstacle with the intent to reach a social goal stimulus (only initially visible).

### 3.2. Experiment 2—Detour Behavior in the Enlarged Four-Compartment Box

**Figure 4:**
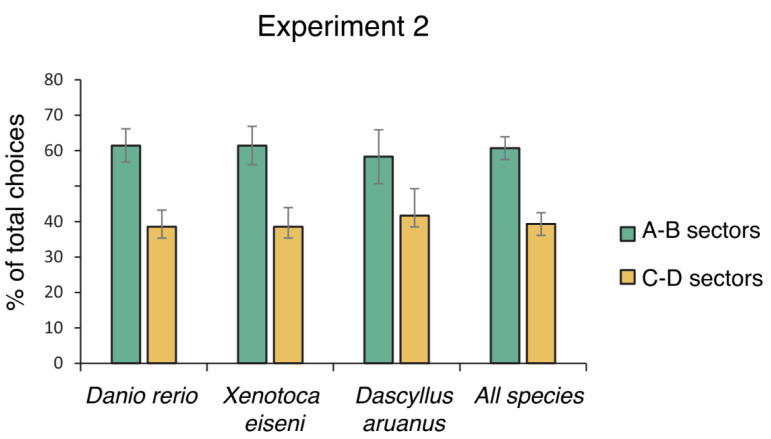
Percentage of total choices (mean ± SEM) for the correct compartments close to the social goal (A–B) and the incorrect compartments far from the social goal (C–D).

Only 1 of the 16 *Dascyllus aruanus* failed to exit the corridor within 10 min for all the test trials, showing a freezing reaction. All the other fish were able to complete the four test trials (Figure 4). The total choices for each compartment visited were analyzed using an ANOVA, considering the different species (*Danio rerio*, *Xenotoca eiseni*, *Dascyllus aruanus*) as between-subject factors and compartments (A–B vs. C–D) and days (1st day vs. 2nd day of test) as within-subject factors. The ANOVA revealed a significant effect of compartments (F(1,60) = 9.579, *p* = 0.003, $\eta_{p}^{2}$ = 0.138), while no difference was found in its interaction with species (F(2,60) = 0.082, *p* = 0.921), day (F(1,60) = 1.878, *p* = 0.176), and among compartments × species × days (F(2,60) = 0.774, *p* = 0.466). Moreover, no statistical differences were found if comparing the two correct compartments or the two incorrect compartments (respectively, A vs. B: t(62) = −0.067, *p* = 0.947; C vs. D: t(62) = 0.271, *p* = 0.788).

On the two freshwater species, further analysis was performed aiming to detect whether performance differed between males and females, showing no difference in accuracy performance related to sex (compartments × sex: F(1,44) = 0, *p* = 1.00; compartments × species × sex F(1,44) = 0.080, *p* = 0.779; compartments × days × sex: F(1,44) = 1.607, *p* = 0.212; compartments × days × species × sex: F(1,44) = 2.511, *p* = 0.120).

Analysis restricted to the first choices revealed no statistical difference among the three species in choosing the correct compartments (A–B) and the incorrect compartments (C–D) (Kruskal–Wallis test: H(2) = 0.988, *p* = 0.610). A χ^2^ test showed a general preference for the correct compartments over the incorrect ones (χ^2^ = 8.397, *df* = 1, *p* = 0.004).

**Figure 5:**
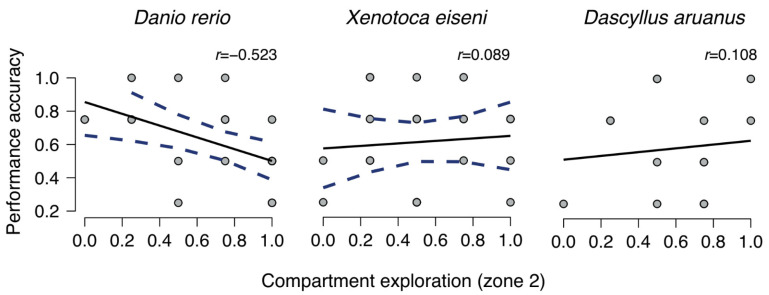
The regression lines for each fish species (*Danio rerio*, *Xenotoca eiseni*, *Dasyllus aruanus*) show the correlation between performance (i.e., “performance accuracy” index) and the behavior inside the compartments (i.e., “compartment exploration” index), where the value 1.0 represents the choice for the zone 2 (a greater distance than the position of social attractors) and 0.0 the choice for the zone 1 (the effective distance of the social attractors).

**Figure A1:**
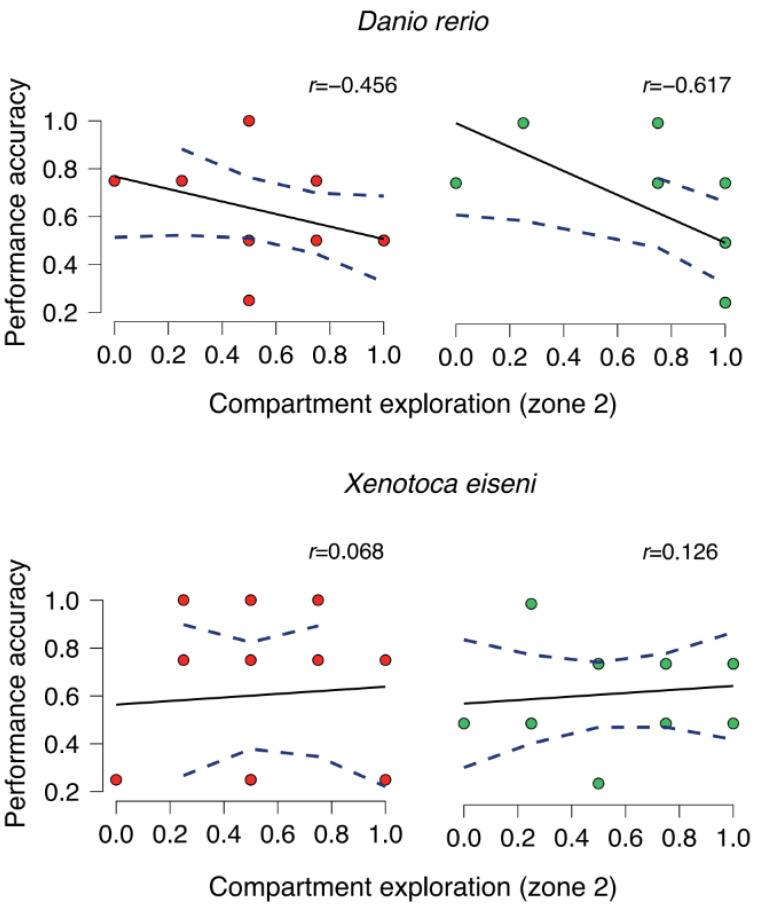
The regression lines show the correlation between performance (i.e., “performance accuracy” index) and the behavior inside the compartments (i.e., “compartment exploration” index) in females (red dots) and males (green dots) of the freshwater species (*Danio rerio* and *Xenotoca eiseni*). The value 1.0 represents the choice for zone 2 (a greater distance than the position of social attractors), and 0.0 is the choice for zone 1 (the effective distance of the social attractors).

Further analysis was performed to check whether the choice of the correct or incorrect compartments correlated both with different exploratory behavior in the chosen compartment (i.e., whether fish entered zone 1 or zone 2) and a different time in making a choice (i.e., time 1 and time 2). To do so, for each subject, an index of “performance accuracy” (that is, number of correct choices for A–B/total of four trials) and an index of “compartment exploration” (that is, number of visits in zone 2/total of four trials) were calculated. A Pearson’s correlation analysis showed a negative correlation between “performance accuracy” and “compartment exploration” in zebrafish (*Danio rerio*) (r(22) = −0.523, *p* = 0.009; males zebrafish: r(10) = −0.617, *p* = 0.033; females zebrafish: r(10) = −0.456, *p* = 0.136), indicating a progressive decrease of exploration in zone 2 as the number of choices for the correct compartments (A–B) increases. However, the same correlation was not found in the two other species (all *p* values > 0.05) (Figure 5; see also Figure A1 of Appendix A for correlations in males and females of *Danio rerio* and *Xenotoca eiseni*).

**Figure 6:**
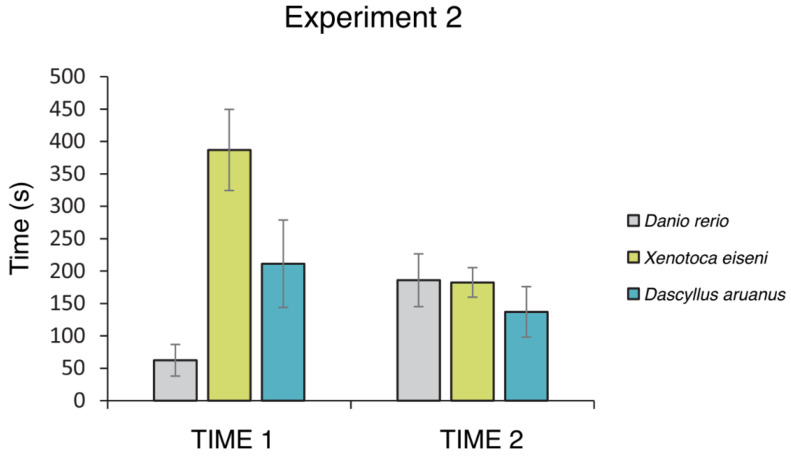
Time needed to leave the observation area (time 1) and time elapsed from exiting the observation area until choosing a compartment (time 2) (group means ± SEM). The left chart reports the performance of the three species observed.

**Figure A2:**
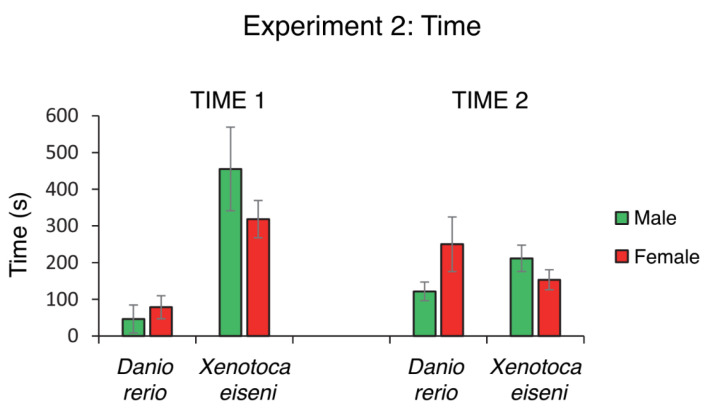
Time needed to leave the observation area (time 1) and time elapsed from exiting the observation area until choosing a compartment (time 2) (group means ± SEM) in females and males of the freshwater species (*Danio rerio* and *Xenotoca eiseni*).

A correlation between “performance accuracy” and both “time 1” and “time 2” showed no significance in any species (*p* values > 0.05 in all groups, see Supplementary Materials). Nonetheless, irrespective of the lack of correlation between accuracy and time, the time needed to leave the observation area (i.e., time 1) was different among species (F(2,60) = 11.009, *p* < 0.001, $\eta_{p}^{2}$ = 0.268). Post hoc analysis revealed that *Xenotoca eiseni* spent more time in the observation area than *Danio rerio* (*p* < 0.001) and *Dascillus aruanus* (*p* = 0.030) (see Figure 6). No significant effect was found in analyzing the time elapsed after exiting the observation area and the choice of compartment (i.e., time 2: F(2,60) = 0.504, *p* = 0.607). Lastly, analysis of the time limited to the two freshwater species showed no difference between females and males of *Danio rerio* and *Xenotoca eiseni* both in time 1 (F(1,44) = 0.610, *p* = 0.439) and time 2 (F(1,44) = 0.596, *p* = 0.444). However, an interaction between species and sex was found in time 2 (F(1,44) = 4.256, *p* = 0.045, $\eta_{p}^{2}$ =0.088) but not in time 1 (F(1,44) = 1.587, *p* = 0.214) (Figure A2 of Appendix A).

Overall, the results of Experiment 2 showed that all of the marine and freshwater species studied seemed able to detour an obstacle with the intent to reach a social goal stimulus (only initially visible), even when required to adopt a larger detour and enter one of four elongated compartments that could extend in the direction of the social group or the opposite direction. Moreover, discerning whether fish behavior was different when the correct or incorrect compartment was chosen was studied by analyzing the depth of exploration in the selected compartment. The results indicate zebrafish (*D. rerio*) exhibit a stronger progressive decrease in the exploration of zone 2 (a greater distance than the position of social attractors) as the number of choices for the correct compartments (A–B) increases than in the other two species (*X. eiseni* and *D. aruanus*), suggesting that the best performance accuracy also corresponds to the choice for the correct distance to the social attractors (zone 1). On the other hand, analyzing the time needed to complete the trial, results revealed another species-specific trait, as redtail splitfin fish (*X. eiseni*) spent more time in the observation area than the other two species (*D. rerio* and *D. aruanus*), the marine species (*D. aruanus*) spent an intermediate time, while zebrafish (*D. Rerio*) spent the least time in the same area.

## 4. Discussion

When exposed to the possibility of rejoining a group of conspecifics not directly accessible, all the marine fish species proved able to circumvent an opaque barrier to reach the previously observed goal. The results showed no differences among species, suggesting that all the marine fish engaged in the detour task used the same strategy. Fish showed a significant preference for the correct compartments both in the first absolute trial (i.e., trial 1, day 1) and in the total trials (i.e., the four trials collected in two subsequent days), revealing that the strategy was spontaneously applied by the fish and maintained among the trials. No learning effect could be applied here since the experimental fish could not reach their social companions nor even see them after moving away from the opaque barrier to circumvent it. The conspecifics visible at the beginning of each trial served only as social attractors, and it can be assumed that fish motivation did not change during the two daily test sessions. These results are consistent with the evidence of previous literature that investigated freshwater fishes in the same experimental conditions [23] and in other detour tasks (e.g., [10,13,33,34,35,36,37,38,39]), suggesting that despite the different environments inhabited by freshwater and marine fish, this fundamental skill is present in both.

Based on the findings obtained, a second apparatus was designed to further investigate detour abilities in aquatic species to verify whether such competence was also maintained even when a larger obstacle circumvention was allowed beyond the barrier in order to cover the effective distance of conspecifics from the barrier. Although a clear preference for the compartments close to the stimulus was found in Experiment 1, the compartments did not expand beyond the obstacle (i.e., the net barrier). To allow this, Experiment 2 investigated whether fish preference for the goal-proximity compartment was maintained even in a larger apparatus, introducing long compartments extending much further than the absolute goal position. The use of such a long compartment was also conceived with the idea of investigating whether the choice for the correct or incorrect compartments was also connected with a different behavioral exploration, as maintaining a precise cognitive representation of the conspecifics’ location and an evident “intention” to reach them.

In general, the results of both marine and freshwater fish species in the four-compartment box task showed their ability to detour an opaque barrier to reach a goal stimulus, even when a larger detour was needed. The evidence from Experiment 2 further strengthens the results obtained in Experiment 1 and those of a previous study of only freshwater fishes [23].

The particular relevance of this second experiment was that all the species studied showed not only their preference in approaching the compartments close to the social stimulus but would do so even when a large detour was required. Moreover, the compartments were located in a deeper position with respect to the obstacles (i.e., the net barrier), and the fish that chose the corridor effectively overcame the net barrier from which they had observed the social group, covering the distance that separated them. This provides further evidence of the willingness to keep the motivation to pursue the goal and a possible lasting mental representation of the goal to be reached even during momentary loss of sight of the goal during the circumvention of the opaque barrier.

The absence of interspecies differences suggests that all the fishes showed the same detour skills, despite inhabiting different environments (i.e., marine and freshwater), having developed similar abilities to circumvent obstacles for facing the same ecological pressures and needs. Nevertheless, even if showing similar accuracy in solving the detour task, differences among species were found relative to the time fish required to abandon the visual goal before performing the barrier circumvention. In Experiment 2, results showed that *X. eiseni* spent more time in the observation area compared with *D. rerio* and *D. aruanus*; the marine species (*D. aruanus*) spent an intermediate time, while *D. rerio* took the least time to leave the same observational area. This might reflect a different level of cognitive mechanisms recruited in regulating the inhibitory control, which allows animals to inhibit the strong tendency for a direct reach of the visible reward behind the barrier [54,55]. Alternatively, such different times spent in the observation area might also be a species-specific characteristic. For example, it could be attributable to the amount and speed of movement of the two species, which is significantly greater in zebrafish (e.g., [56]), while slower redtail splitfin (*Xenotoca eiseni*) stay longer in the observation area and delay the exploratory activity. Although the three species were observed to take different amounts of time to leave the observation area, this was not correlated to the accuracy in detouring and choosing the correct compartments, which is not different among the three species observed. Therefore, the observed difference does not seem attributable to the native habitat (freshwater vs. marine water) but rather to a characteristic of the single species.

Another interesting result that emerged was that zebrafish (*Danio rerio*) showed a different behavioral exploration in the compartments (zone 1 vs. zone 2) when the correct (A–B) or incorrect ones (C–D) were chosen relative to the other two species. When choosing the correct compartment close to the goal, zebrafish (in particular, males) showed a higher exploration focused on the central zone of the selected compartment (zone 1), corresponding to the effective distance of the social attractors; and when the “incorrect” compartment was approached, they mainly swim to the end of the compartment (zone 2), corresponding to a greater distance than the position of social attractors. Since zone 1 was in the midline of the compartment and at the same depth level as the social conspecifics previously observed from the observation area, this might support the conclusion that fish were actively searching for the goal (probably calculating the exact distance to search) and, once not encountered where it was supposed to be, they decided to go back to the starting position instead of moving far from it. On the other hand, the few choices for the incorrect compartments were mostly associated with a swim directed to the end of the compartment, suggesting that these choices were possibly driven by an interest to find a shelter rather than an active search for the goal or, again, by an attempt to reach social companions on the move, who in the meantime have moved further away from the observer. However, this interesting correlation between behavior accuracy and choice for the effective distance of social attractors was found mainly in zebrafish, suggesting that different exploratory activities are present among species, perhaps related to their particular ecology [13]. One hypothesis of such difference could be attributed to a stronger shoaling motivation in zebrafish [57], and this peculiarity has been also used as a social reward to investigate several cognitive abilities (e.g., numerical abilities: [58,59,60]; spatial reorientation: [56,61,62,63,64,65]; amodal completion: [66]; lateralized behavior and asymmetries: [46,50]).

Differences between sexes were taken into consideration only for the two freshwater species, where sexual dimorphism is present and is more accentuated in redtail splitfin fish (*Xenotoca eiseni*) than in zebrafish (*Danio rerio*). No relevant sex differences in detour skills were found in this study, neither in the success of the detour task nor in the time needed to leave the observation area. It may be mentioned that zebrafish males and redtail splitfin females showed less time elapsed from exiting the observation area to choose a compartment, perhaps driven by different motivations between sexes—schooling for females and mating for males (the social goal consisted of four females in the sexed species), assuming that the schooling motivation for females could be more dominant in redtail splitfin fish and the intention to seek a mate could be more dominant in zebrafish. Similarly, zebrafish males showed enhanced accuracy compared to females in choosing zone 1, corresponding to the effective distance of social attractors in relation to the choices of the correct compartments after circumventing the barrier. Interestingly, in the study by Triki and Bshari [40] on detour abilities in the marine sex-changing cleaner fish *Labroides dimidiatus*, a protogynous hermaphroditic species, where all males have previously been females, females showed enhanced abilities in a detour task, consisting of bypassing a barrier to reach a food reward. Thus, it is likely that even the differences between sexes in individual species reflect intra-specific peculiarities not necessarily linked to habitat characteristics alone.

In detour tasks with an initially visible goal, it has been proposed that animals need to create and maintain a mental representation of the non-visible goal throughout the detour route [15,26]. The effective need to represent and maintain a cognitive mental representation of the goal for detouring the obstacle has been argued by Walker and Miglino [29]. Using simulated robots, the authors showed that artificial organisms could make simple detours based on the inputs detected by the proximity sensory feedback without any internal representation of the goal, instead using primitive exploratory behavior such as taxis and wall-following [29,30]. However, the different exploration patterns between correct and incorrect compartments found in Experiment 2 in zebrafish, as well as the tendency to not explore further than zone 1, cannot be exclusively explained using the parsimonious hypothesis of only relying on primitive exploratory behaviors. Instead, this might provide evidence in favor of an effective understanding of the “detour problem” and the real intention to reach the goal, suggesting the persistence of social object retention. From this point of view, the second apparatus represents an improvement compared to the first, given that fish can attempt to locate the shoal by swimming to an approximate position, strongly suggesting the fish are keeping the shoal in mind and not just relying on taxis.

## 5. Conclusions

After observing a group of social companions from a grid without the possibility to directly approach them, all the marine fishes considered proved able to move far from the goal, lose visual contact with it, detour the obstacle, and finally approach the compartment in proximity to the goal. This is the first evidence of a detour task in marine fish species using a four-compartment box task with an opaque barrier, social attractors, and without a learning procedure. Moreover, the present study has shown that both marine and freshwater fishes maintain a preferential detour toward the goal object, represented by a group of conspecifics, even when it requires a large circumvention of the obstacle, showing an active attempt to reach the no longer visible social stimulus beyond the obstacle (i.e., the panel with the grid from which the social attractors were visible at the beginning of each trial). The comparable performance to that of the freshwater fish species suggests that the selective ecological pressure was similar across the different aquatic habitats (i.e., sea and freshwater basins). The marine species observed here might have developed detour solutions due to the presence of obstacles and shelters; in fact, those fish naturally inhabit areas in the proximity of coral reefs. On the other hand, it would be interesting to compare our results by extending the investigation to marine species inhabiting open-sea or ecological niches totally lacking natural features to evaluate in more detail the impact of the environment and detour opportunities on the behavioral habits of species.

Finally, the different exploratory behaviors observed among correct and incorrect compartments (particularly in zebrafish) might provide further evidence in favor of a possible mental representation of the social goal stored in the working memory while performing the obstacle circumvention. Therefore, an alleged “permanence in existence” of the social object might exist, as opposed to a parsimonious hypothesis suggesting that the detour ability emerges only from primitive forms of exploratory behavior such as taxis and wall-following. Detour ability, with its related cognitive skills, seems to constitute basic survival equipment, suggesting object persistence may play a role as a possible interesting element for the intelligent problem solution.

## Data Availability

The original contributions presented in the study are included in the article/Supplementary Materials, further inquiries can be directed to the corresponding author/s.

## Supplementary Materials

The following supporting information can be downloaded at: https://www.mdpi.com/article/10.3390/ani14172572/s1, Excel Data File: detour supplementary material.
